# Patient engagement in fertility research: bench research, ethics, and social justice

**DOI:** 10.1186/s40900-021-00278-x

**Published:** 2021-05-12

**Authors:** Perry R. Fleming, Makayla M. Swygert, Coen Hasenkamp, Jessica Sterling, Ginny Cartee, Rebecca Russ-Sellers, Melanie Cozad, Renee J. Chosed, William E. Roudebush, Ann Blair Kennedy

**Affiliations:** 1 Clemson University, Clemson, South Carolina USA; 2 University of South Carolina, Patient Engagement Studio, Columbia, South Carolina USA; 3 University of South Carolina, School of Medicine Greenville, Columbia, South Carolina USA; 4 University of South Carolina, Arnold School of Public Health, Columbia, South Carolina USA; 5 Prisma Health, Family Medicine, Greenville, South Carolina, USA

**Keywords:** Patient participation, Stakeholder participation, Research personnel, Reproductive techniques, RNA sequence analysis, Trust, Fertilization in vitro, Surveys and questionnaires

## Abstract

**Background:**

Patient and Public Involvement (PPI) in research is increasingly being utilized to better connect patients and researchers. The Patient Engagement Studio (PES) supports PPI in research by working directly with researchers throughout various stages of their projects. Recently, two researchers presented to the PES for assistance with their project, Embryo+™. The purpose of Embryo+™ is to decrease miscarriage rates using RNA sequencing technology that screens for the most viable embryos. To date, no examples of PPI directly in the planning or implementation of bench research concerning in vitro fertilization and embryo transfer have been identified.

**Main body:**

Embryo+™ researchers met in-person with the PES two times (fall 2019; each meeting had 9 PES members in attendance) for initial feedback and protocol development. After these meetings, PES leadership and Embryo+™ researchers decided that the unique nature of the project merited a PPI evaluation. Subsequent evaluation of engagement efforts occurred by reviewing the PES reports for the Embryo+™ researchers, conducting two recorded web-based discussion meetings with the PES (summer 2020; meeting 1 *n* = 7; meeting 2 *n* = 6), and a brief survey (*n* = 13). The discussion meetings provided an opportunity for the PES members to define engagement themes through consensus via verbal agreement to the studio director’s periodic summaries during the discussions. Combining survey results and PES themes allowed for a broad discussion for meaningful engagement.

The Embryo+™ researchers established trust with the patients by changing some of their language in response to patient suggestions, allowing for unintended ethical conversations, and implementing the patient developed protocols. Overall, the patient experts thought this project was very meaningful and valuable, quantified by a mean loyalty score 89.43 (s.d. 10.29).

**Conclusion:**

Bench science researchers may need additional PPI training prior to engaging with patient groups. PPI in this project was successful in large part due to this training, where the director emphasized the importance of gaining trust with the patients. The researchers applied what they learned and several examples of how to develop trust with patients are discussed. If trust is established, PPI in an ethically charged, basic science research study can be both valuable and successful.

**Supplementary Information:**

The online version contains supplementary material available at 10.1186/s40900-021-00278-x.

## Plain English summary

Patients are increasingly becoming involved in all stages of the research process. Patient and public involvement has been shown to answer questions that matter to patients, increase enrollment in studies, and improve the spread of research results to the public. However, there are limited evaluations of patient engagement in basic science research (research performed in a laboratory setting in various fields). Here, we provide an example of patients effectively involved in the planning and implementation of an ethically complex study in the field of Assistive Reproductive Technology. A Patient Engagement Studio, affiliated with an academic health center, directly connects patients and researchers through bi-monthly meetings. Recently, two researchers presented their project, Embryo+™, to the studio during the planning stage of their study. This project aims to use a new testing technology to reduce miscarriage rates. The researchers presented to the studio twice (fall 2019), and two follow-up meetings were conducted with the patients (summer 2020). Also, the patients completed a survey evaluating how engaged they felt with the project. Through the meetings, the researchers changed their language in response to patient feedback, and patients developed project protocols. Survey results showed that the patients thought this project was very meaningful and valuable. Overall, this evaluation shows that patients can add value to contentious bench science projects, particularly in the field of Assistive Reproductive Technology.

**Supplementary Information:**

The online version contains supplementary material available at 10.1186/s40900-021-00278-x.

## Background

The term “bench science” is often used interchangeably with “basic science research” and primarily encompasses research performed in a laboratory setting in various fields (Biochemistry, Pharmacology, Immunology, etc.) [1]. While patients are increasingly becoming involved in all phases and disciplines of research, few studies are reporting Patient and Public Involvement (PPI) in basic science research [2]. PPI in other research areas is well documented in the literature and has been shown to answer questions that matter to patients, increase enrollment, reduce attrition, and improve dissemination [3–5]. It is most likely that the lack of PPI in bench science is due to various perceived challenges such as identifying the patient’s role [6], redefining long-lasting research hierarchies [7], and prioritizing ethical issues such as justice [2].

A potential bench science field where PPI could be particularly beneficial is Assistive Reproductive Technology (ART). Patient-centered research involving ART, including in vitro fertilization and embryo transfer (IVF-ET), is often centered around ethics, public policy, or individual motivation and perspectives for initiating or discontinuing treatment. Our literature review did not uncover any instances of PPI directly in the planning or implementation of bench research concerning IVF-ET. This apparent deficit concerning the integration of PPI into the research process may result in research outcomes that are less beneficial to patients. In addition, bench researchers may remain disconnected from the direct needs of the patients who will ultimately benefit from their research.

Recently, ART researchers contacted a Patient Engagement Studio (PES) regarding their ethically complex study, Embryo+™. With funding organizations increasingly requiring PPI, in order to reduce the tokenization of PPI and to determine if the engagement with patients on a project has been meaningful, it is important to review and evaluate the engagement process [8]. Additionally, it is also important to determine the impact of the engagement on the study [9]. Therefore, the purpose of this paper is to provide an example of how to integrate and evaluate patient and stakeholder engagement in the early stages of the Embryo+™ project, an ethically complex bench science project in the field of ART.

### Embryo+™ project

Infertility is a disease that affects both men and women. The CDC National Survey of Family Growth reports that 13.1% of women have impaired fecundity, and 12.7% have sought infertility services [10]. Couples often seek ART for fertility treatments such as IVF-ET. Despite continued innovation in these technologies, IVF-ET still attracts a high price tag that can limit patient access to this technology as well as limit the number of embryo transfers patients can afford. In addition to its high economic barrier, a successful pregnancy carried to term is not guaranteed as the live birth rate per egg retrieval with IVF-ET is approximately 55% for women under 35 but drops to 4–13% for women over 40 [11]. The economic and documented psychological burden [12] imposed on patients utilizing IVF-ET creates a need for improved screening of IVF generated embryos before transfer to increase the likelihood of a successful pregnancy.

To meet this need, Embryo+™ is a proposed clinical research trial that plans to utilize RNA sequencing (RNAseq), a sequencing technology, on blastocoel fluid from IVF-generated embryos to assess gene expression to identify the most viable embryo for uterine transfer. Therefore, the goal of this trial is to enhance subsequent embryo implantation rates, via reduction of miscarriage rates, of infertile patients undergoing IVF-ET. The improvement of implantation rates will result in more term pregnancies in a shorter time, thus potentially lowering the patient’s financial, psychological, and emotional stress.

Specifically, the Embryo+™ researchers approached the PES to seek patient input on the framing of their research questions and to determine a patient-centric process for identifying potential egg and sperm donors to create the embryos for the initial study. Gamete and embryo donations are common and routine in the IVF community. The genetic analysis of these donated embryos is often not known as these tend to be ‘extra’ or ‘surplus’ embryos from a couple’s own IVF process that they have chosen to donate to others trying to conceive. Therefore, the proposed Embryo+ project aimed to generate embryos from gametes (from sperm and egg banks) where many of the donor characteristics are known. The Embryo+ researchers would then perform additional molecular tests on these generated embryos to select the most viable embryo for uterine transfer. The PES was asked to assist in developing a protocol for selecting which gametes to use for the generation of these embryos.

### Patient engagement studio

The PES is a diverse group of patients, researchers, clinicians, and public stakeholders affiliated with an academic health sciences center. The mission of the PES is to meaningfully integrate the patient voice in all stages of research by promoting collaboration with scientists and clinicians to optimize health and research outcomes. The PES collaborates with research teams in planning, conducting, and disseminating research results and health system innovations and assisted with the planning stage of the Embryo+™ study.

To clarify, within the PES we use the term “Patient Expert” to denote expertise by our patient members. The Patient Experts have participated in training on team building, research methods, and communication. These individuals are also experts at being patients within a healthcare system. To become a Patient Expert, individuals must have had extensive experience with a health condition that has caused significant interaction with the healthcare system and/or be the caregiver of a similar individual. While there are eight to twelve regularly participating Patient Experts, “guest experts”—those who have the condition being studied or are a part of the research teams’ participant population—are at times invited to attend as well. For example, if a study is being planned that includes pregnant women or adolescents, these individuals are often invited to participate as they have unique experiences which may be overlooked if they are not included.

The PES has scheduled meetings on the first and third Tuesday of each month for an hour and a half, with the Patient Experts being compensated for their time. Currently, we compensate each Patient Expert $50 for each meeting. The studio normally meets face-to-face; however, meetings have taken place virtually since March 2020 due to the global COVID-19 pandemic. The meetings with the Embryo+ researchers were conducted in person within a classroom space in the nursing school building on the health system’s main campus.

**Fig. 1:**
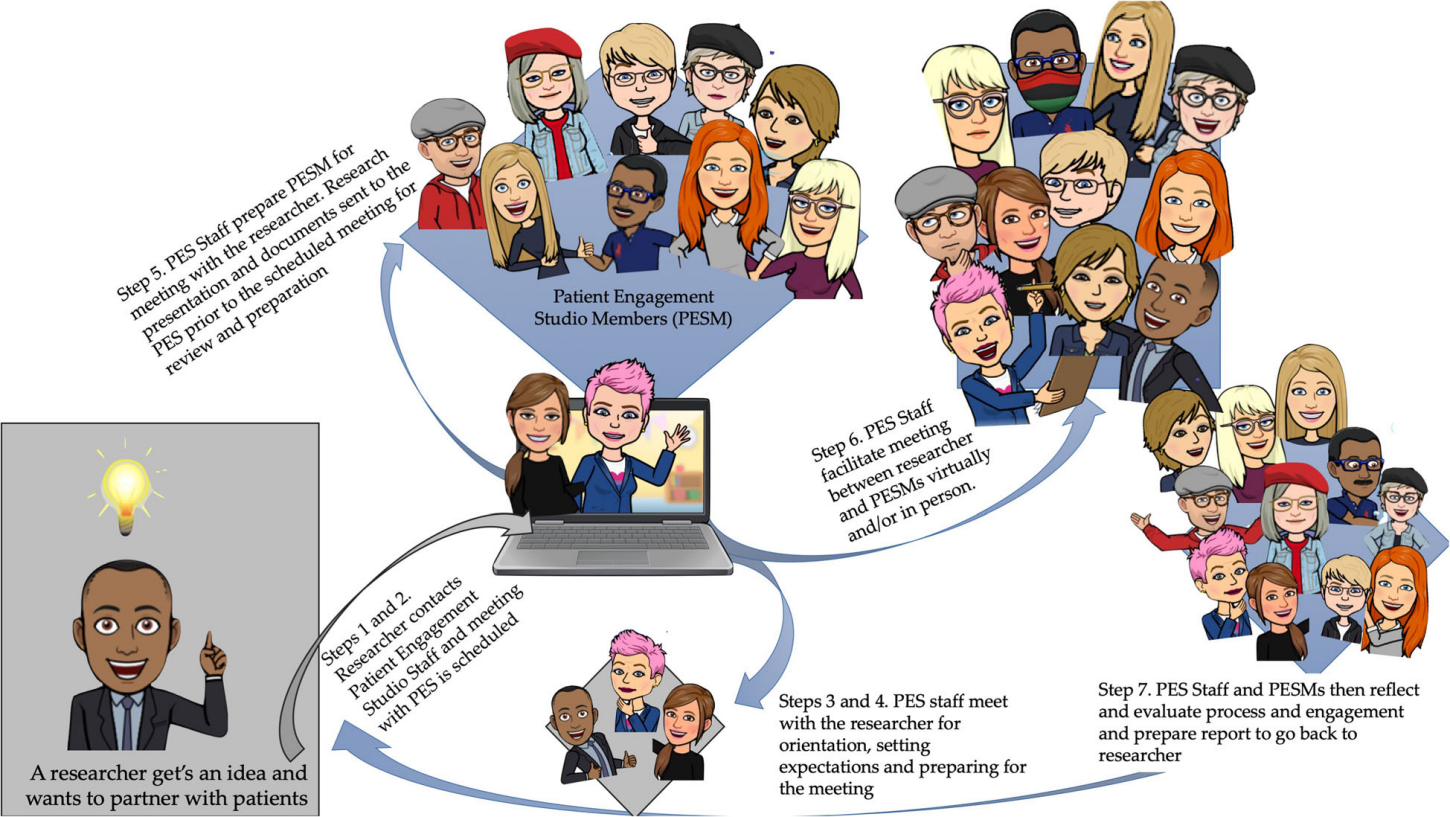
Seven step process for engaging with the Patient Engagement Studio

The PES has identified a seven-step process (Fig. 1) for researchers who wish to engage with the group: 1) researchers contact the director of the PES to discuss meeting expectations; 2) researchers schedule a time to meet with the PES members; 3) the director then provides researchers with a Patient Expert-created PowerPoint template for the presentation that focuses on communicating their study to the patient audience; 4) the director reviews the researcher’s presentation prior to the scheduled meeting to assess the presentation for patient-centeredness and provide feedback; 5) researchers send the presentation and any documents to the PES several days prior to the scheduled meeting for review and preparation; 6) researchers meet with the PES; 7) the PES Director and Staff create a report summarizing the meeting and provide it to the researchers. This report is based upon the Patient-Centered Outcomes Research Institute (PCORI) engagement rubric. The aforementioned Patient Expert-created PowerPoint template and the report template are provided in Additional file 1: Appendix A.

### PPI evaluation method

ART researchers sought the advice and counsel of patients and other stakeholders from the PES during the development of their research question and proposal, stages 1 and 2 in the CORE Research Stage Framework [9]. The researchers met in-person with the PES twice for feedback on their project 1 month apart in the fall of 2019. At both of the meetings, nine Patient Engagement Studio Members (PESMs) were in attendance at each meeting, however only five attended both meetings. During the meetings PES staff took notes capturing PESMs comments and questions; these notes are then used to prepare feedback reports for the researchers. In this case, each report was approximately 4 pages in length, single spaced. It should be noted that two of the PESMs had used IVF, and one had fertility issues and adopted a child.

Upon reflection after the first two PES meetings, the studio director determined that the unique nature of the Embryo+™ project, both ethically complex and from the ART field, merited an internal PPI evaluation. The director brought on two PES interns to assist in the evaluation. To evaluate engagement, PES interns subsequently conducted two virtual follow-up discussion meetings with the PES in summer 2020 to explore and reflect upon the PESMs experiences with the Embryo+™ researchers and their perceptions of the fall 2019 meetings. Additionally, a brief survey (Additional file 2: Appendix B) was created in Qualtrics [13] and sent electronically to the PESMs who attended the original meetings to gather demographics and perceptions of engagement with the project quantitively.

The interns prepared for the discussion meetings by reading both feedback reports and having conversations with the studio director to better understand the emotional and dynamic context of the first two meetings. They developed questions and tailored discussions based on the CORE measures (patient-centered, meaningful, ethical and transparent, etc.) [9] and GRIPP2 [14] reporting checklist (discussion question are in Additional file 3: Appendix C). The interns asked the PESMs questions about how they felt about the researchers and their presentations, what they remembered about the meetings, and what they believed were the researchers’ goals in coming to the PES for feedback. Both discussion meetings were held using a video conference format and recorded. At various points throughout the meetings, the director would summarize the discussion and then ask for consensus from the group. Consensus was determined by verbal agreement and physical actions (e.g. nodding, giving a thumbs up) of the PESMs. After the discussion meetings, the interns and the studio director debriefed and developed main takeaways based on the group consensus of the PESMs’ experiences and perceptions.

For the survey, likert scale questions asked specifically about the PESMs’ satisfaction with their participation in this project (strongly disagree to strongly agree), if the project was a good use of time, their likelihood of continuing to work with the researchers, the likelihood of them recommending to others that they become a PESM or a participant in Embryo+™, and how valuable they found their participation in this project. These final four questions were combined into a composite loyalty variable to determine the participants’ loyalty to the project [15, 16]. Loyalty measures provide a more robust finding than satisfaction measures, as satisfaction is identifying an attitude, and loyalty includes attitudes and behaviors [15, 16]. The PES uses this score as a proxy to understand if patients feel meaningfully engaged with that project. We posit that if patients report higher levels of loyalty to a project, they may therefore be reporting that the engagement is meaningful. In the future this measure needs to be validated as a meaningful engagement measurement. Each of the responses in the four questions was transformed into a 100-point scale, and the composite variable was calculated by averaging the responses together. Frequencies and percentages were calculated in Qualtrics survey software [13]. Once the results from the discussion meetings and the survey were collected and summarized, the interns and studio director then discussed how these results fit within the CORE framework to allow for greater understanding of engagement with the project.

### Participants

The PESMs who engaged with the bench science researchers were not study participants nor affiliated with the Embryo+™ project. These panel members comfortably share their identities, and the demographics of those who participated in the Embryo+™ meetings are listed in Table 1.

**Table 1:** Patient Engagement Studio Demographics–Embryo+™ Participation

Variable	n = 13
Age, mean (SD) min, max	58 (14.08) 35,73
Gender identity, n(%)	
Man	3 (23.8)
Woman	9 (69.23)
Transgender woman	1 (7.69)
Race/Ethnicity, n (%) (% of cases)	
Black or African American	2 (15.38)
White	10 (76.92)
Choose not to answer	1 (7.69)
Hispanic/Latino/a/x	0 (0)
Patient Engagement Studio Role, n (%) (may check more than one role)	*n* = 18
Patient Expert	11 (61.11)
Community member	1 (5.56)
Clinician	1 (5.56)
Staff member	1 (5.56)
Researcher	4 (22.22%)
Meeting attendance, n	
Embryo+™ meeting 1 (project introduction)	9
Embryo+™ meeting 2 (selecting donors)	9
Discussion about meeting 1	7
Discussion about meeting 2	6

#### Meeting 1

Prior to the first meeting, PESMs reviewed the established meeting ground rules and received a copy of the PowerPoint that was to be presented. The PESMs were advised that the ethics of IVF as a treatment and embryo screening would not be discussed at this meeting to provide time to focus on clarifying research questions and developing a protocol for the selection of gametes to use in the generation of embryos. It should be noted that there is extreme value in including PPI in discussions about and around potential ethical issues in research [17–19]. However, given that there was a limited meeting time and many ethical issues surrounding IVF as a treatment have been addressed by medical ethicists, discussions were aimed at focusing on the meeting agenda.

In the first meeting, the Embryo+™ researchers began by describing the topic of miscarriage and its relation to IVF-ET. They discussed the cost of IVF-ET with preimplantation genetic analysis in the United States (on average, $23,000 per cycle) [20]. The researchers then went on to explain Embryo+™ and its goals and sought input from the PES as to whether this would be a meaningful project for patients. The PESMs indicated in this meeting that the project was very meaningful to the patient community as the project seeks to directly lower miscarriage rates in IVF-ET patients, thus reducing their physical, emotional, financial, and psychological stresses by allowing them to have a better chance at a successful pregnancy and childbirth.

##### Clarifying the research question

During this meeting, the PESMs provided feedback to clarify the research question and to minimize disruptions to future study participants. An important topic that was discussed in this meeting was the terminology that the researchers used when talking about IVF-ET. For example, the PESMs suggested that the researchers refer to embryo “viability” rather than embryo “quality” and use “developing” or “generating” an embryo as opposed to “creating.” Additionally, one of the PESMs stated that she preferred saying they were selecting the “embryo most likely to result in a healthy child for the couple.” This feedback was very impactful to the Embryo+™ researchers as they stated that they had not considered the importance of language when discussing embryo research with patients and adapted their vernacular directly because of the PES’s feedback. PESMs and researchers felt these changes would lead to more acceptance of Embryo+™ by patients who would be enrolled in future studies validating its effectiveness on miscarriage and pregnancy rates.

##### Ethical dilemmas and social justice

As previously mentioned, the original purpose of bringing the Embryo+™ project to the PES was not to have an ethics discussion; however, the PES believed it was necessary to have these discussions in order to provide quality feedback to the researchers. Through discussion, it was recognized that the selection of gametes by the PESMs for the generation of embryos had ethical dimensions beyond the original choice to undergo IVF/embryo screening. The PESMs discussed the ethical issues surrounding both IVF and more specifically the selection of gametes for the generation of embryos. These issues mostly centered around the autonomy of the future child and also the social justice implications.

Some of the PESMs expressed discomfort around aspects of IVF itself, which would arise whether or not pre-implantation testing was performed. For example, they worried their feedback could lead to the “creation of designer babies” or that they were “playing God.” While some of the PESMs worried that patients may use Embryo+™ to create a designer baby, the researchers explained that most patients are looking for health information such as predisposition to Alzheimer’s Disease or the presence of the BRCA gene. The PES emphasized the need for an ongoing ethics committee and PES involvement while conducting this research. Some of the additional concerns of the PES included: What if a white couple wants to have a baby of a different race/ethnicity? What if siblings are created and happen to meet 1 day and have a relationship? If patients try Embryo+™ several times and it is not successful, do they get their money back? Another question that could be considered by the PES in the future specifically centers on child autonomy. Is a child’s autonomy violated by parents making choices with this technology on their behalf? If pre-implantation screening did not exist, then this would not be an issue. However, since the technology is available, it will be used, and the future offspring may rank health implications differently than the parents.

Additionally, there was much discussion among the PESMs regarding the cost of the service. The PESMs noted that while the proposed price of Embryo+™ is high, it is less expensive than current IVF-ET treatments or adoption. Nonetheless, the PESMs agreed that the high cost of IVF-ET excludes a portion of the population who is unable to afford the costs of treatment. One of the PESMs expressed concern that this sends the message that certain people should not have children, or that they should only be allowed to have “bargain-basement children.” They worried that this conveys the message that if patients have the money to afford IVF-ET, then they are “worthy” of being parents, while those who cannot afford IVF-ET are not worthy. To alleviate some of these concerns, one PESM suggested a sliding scale method for determining cost. The PESM suggested using either the patient’s household income or the embryo “quality” (likelihood of successful implantation) to determine the cost. Many PESMs agreed that a sliding scale based on income was more palatable than a cost that is based upon embryo “quality” and that it allowed IVF-ET to be accessible to more people, thus providing a potentially better outcome for everyone. However, some PESMs worried that the sliding scale method could lead to discrimination both against embryos and against couples seeking IVF-ET. Ultimately, during the first meeting with researchers, this conversation had to be put on hold because the PESMs could not reach a consensus.

#### Meeting 2

The second meeting, held 1 month after the first meeting, sought the PES’s input in determining a process to identify potential egg and sperm donors to generate the embryos. The PESMs selected donors from a company identified by the research team that provided both egg and sperm donors. The researchers requested that for this study, since a majority of the individuals who seek IVF-ET are white, the PESMs start only with white donors. Then, the PESMs were given an overview of the donor company’s website and told that certain features and/or components could be used to help filter out possible donors. It was determined that the group would first select the sperm donor.

##### Process for donor selection

Through an iterative process, the researchers showed the PESMs how the website could be filtered to help choose certain aspects of interest to reduce the number of individuals listed as potential donors. When first launching the website of potential sperm donors, the PESMs saw pictures of the sperm donors when they were children. Some of the PESMs joked about choosing the child that looked the “most studious” or “most gregarious.” However, based on the previous ethical discussion about the potential of “designer babies,” the PESMs determined that they did not want to choose a donor based on looks. They discussed that the most important objective was to help generate an embryo that could result in a healthy child; therefore, their first concerns were health history and genetic abnormalities. They also wanted to pick a donor that would lead to embryos that would be viable for most mothers to carry. To this end, there were discussions specifically about donor blood type and cytomegalovirus (CMV) status. Citing concerns about possible Rh incompatibility and other blood type incompatibility leading to possible miscarriages [21, 22], it was determined that it would be best to select a donor who was O- blood type. CMV status may also impact miscarriage rates [23]; therefore, it was determined that choosing a CMV negative donor was preferred. Health histories and genetic risk factors were also reviewed.

**Fig. 2:**
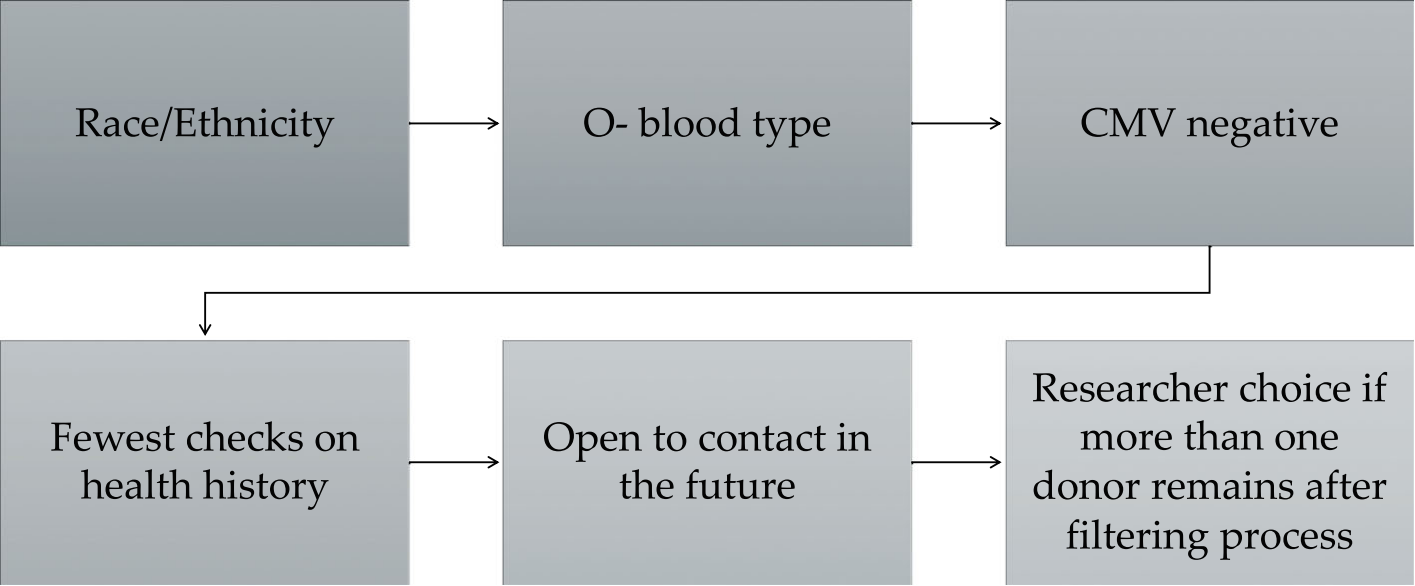
Final process for filtering donors. PESMs determined that first race/ethnicity would be selected, then O- blood type, CMV negative, donor openness to contact in the future by the resultant child, and finally the fewest checks in health history and/or genetic abnormalities. If more than one donor was left, then the researcher would choose the final donor

Prior to beginning the selection of the egg donor, the director summarized the process for filtering donors that had just been created, so that the same process could be applied for the egg donors. The process entailed choosing race/ethnicity, blood type, CMV status, health history and genetic abnormalities. Upon opening the website for egg donors, PESMs saw that the website showed pictures of the egg donors as adults rather than showing baby pictures like were shown for the sperm donors. The PESMs found this very odd. Some of the PESMs wanted to exclude women for being a slightly higher weight (although within average guidelines for women). However, it was decided that the same process would be applied as was used for the sperm donors. Upon applying this process, two egg donors remained. At this juncture, the PESMs noted an additional option. Donors could indicate if they were open to being contacted by the resultant child in the future. The PESMs wanted the child to have the option to contact the donors; therefore, they determined this was a necessary filter for the donors and was slightly more important than health history. The PESMs indicated that after applying this process, if more than one donor remained, the researchers would pick between the final selections. The finalized process for selecting donors is indicated in Fig. 2.

### Meaningful engagement

The Embryo+™ researchers are moving forward with their project. Since the project is only moving into the third stage, not all levels of engagement are yet measurable. However, the PESMs feel it is important to evaluate and share their current experiences and processes as this project is somewhat unique due to the nature of the project and the ethical discussions surrounding it.

#### Qualitative feedback

To determine if the PESMs felt their engagement was meaningful, PPI was evaluated during the follow-up discussion meetings and in the follow-up survey. Meaningful engagement is important to the PESMs. One of the PESMs shared that not all researchers who bring their projects to the PES made them feel heard. During the follow-up meeting, one of the student researchers inquired as to what makes the PESMs feel heard. A PESM stated that it had to do with the researchers’ mannerisms. He felt that some researchers try to answer questions before the PESMs can finish talking, and some researchers cut them off completely. He said that some researchers look at the PESMs like they cannot wait for them to stop talking, so they can tell them the “right way” to think or to approach the project. Another PESM said that it has to do with body language and other non-verbal communication. One PESM felt like some researchers come to the PES for the PESMs to agree with them, not to disagree with them. He appreciated when there is an open dialogue and a back-and-forth conversation between the researchers and the PESMs. The PESMs made sure to express that this was not the way they felt about the Embryo+™ researchers as the researchers made them feel appreciated, heard, and truly involved in the discussion.

The adaptation of the researchers’ terminology at the recommendation of the PESMs provided enhanced meaningfulness for the PESMs. One PESM described feeling an “evolution” between the first and second meetings in terms of a change of mindset and terminology by the researchers. Another PESM stated that it was “powerful” to see the new language in action. She said that the researchers did not fumble when using the new language; this impressed the PESMs and let them know that the researchers had truly made a change in the way they were thinking about the language in their project. One PESM expressed that it made her feel like the researchers valued the PESMs.

The PESMs also acknowledged that while the researchers had asked them not to discuss the ethical implications of the study, there was considerable concern among the PESMs about the moral and ethical components of the project. The researchers did not dismiss these discussions, allowed them to continue, and did their best to assuage the concerns of the PESMs. These discussions appeared to enhance the trust between the PESMs and the researchers. Additionally, they brought to light ethical questions and social justice concerns that the researchers had not yet considered.

Another conversation of particular importance was the discussion regarding the visual representation of the potential gamete donors (e.g., baby pictures for the sperm donors; adult pictures for the egg donors). Several of the PESMs were bothered by the difference between pictures of the egg and the sperm donors. One of the PESMs expressed that she felt like there was a disparity between the photos. She liked the use of baby photos for the men but did not understand why they had used “glamor shots” for the women. She remembered their conversations surrounding the egg donors being more subjective; specifically, she recalled discussions about their levels of fitness or occupations. They had conversations about the women that they had not had about the men. For example, there was one egg donor who already had a child, and there was a discussion about whether she should have even been on the donor website or not. Another PESM expressed that she felt the difference between the egg and sperm donor photos exemplified a larger societal issue of how people think about men and women in their daily lives. She felt the difference was in society’s expectations of what is ok to say about women or what is not ok to say about men. She also felt that it made it more difficult to make an objective decision about the egg donors and that it made the conversation uncomfortable at times. In addition to the photos, a PESM pointed out that some of the information included about the donors in the biographies differed for the egg and the sperm donors. For example, the weight of the egg donors was included, while the weight of the sperm donors was not. Despite these concerns, the PESMs seemed to feel that they were as objective as they could have been in their choice of an egg and sperm donor. The PESMs did recognize that these issues were not the fault of the researchers but rather of the company that provides the gametes. The PESMs suggested that the researchers contact the company and provide them with the feedback from the PES with hopes that they consider the potential implications of providing different pictures and information for the egg and the sperm donors.

#### Survey results

Finally, survey results indicate that all participants agree or strongly agree that they are satisfied with their participation with the Embryo+™ project and that their engagement with the project is a good use of their time. All participants are also extremely or moderately likely to continue working with the Embryo+™ project if researchers continue to engage with the PES. There was greater variation in the likelihood of patients recommending that others join Embryo+™ as a PESM, with three (23.07%) participants indicating that they were either slightly likely or neutral. All others (*n* = 10, 76.92%) were moderately likely or extremely likely to recommend to others that they join as a PESM. Only two (15.39%) of the PESMs were neutral or slightly likely to recommend to others that they become a study participant, with 84.61% being moderately or extremely likely. All participants found their participation with the project to be highly valuable. Loyalty was calculated with a mean of 89.43 (s.d. 10.29).

These descriptions, feedback, and survey results speak specifically to and provide qualitative and quantitative evidence for the CORE measures of team collaboration, patient-centeredness, meaningfulness, understandability, rigorousness, and integrity/adaptability [9]. With continuous use of the CORE measures as a framework for evaluating engagement in this project, a review of the COREs not yet met or measured can allow for more rigorous data collection and evaluation methods in the future. Additionally, PES staff can identify specific items and activities for the Embryo+™ project research team to strive to include in their project to enhance engagement. For example, PES staff could suggest sending any relevant study materials to be reviewed by the PESMs to help support the CORE measure of being ethical and transparent. Providing specific, actionable guidance to a research team may be an essential element needed for those scientists not trained to include PPI in studies. With regards to the Embryo+™ project, provided the researchers continue to engage with the PES throughout the research process, it appears that meaningful engagement may be achieved for this study.

### Lessons learned, limitations, and changes implemented

As PPI continues to be incorporated into various research areas, researchers from disciplines such as bench science may need additional training. In this project, PPI was successful in large part due to PPI training for the researchers during the initial meetings with the studio director. In these meetings, the studio director was able to clarify meeting expectations, give feedback on the prepared presentation, and emphasize the importance of establishing trust with the patients. When the Embryo+™ researchers first began discussions with the studio director, they were not aware of the importance of establishing trust with the patients. However, through these meetings, the researchers were better able to understand its importance for PPI. They applied their knowledge by allowing for ethical conversations even when it was not the focus of the project and by enabling patients to direct the process for designing some of the research protocols (selecting the donors). Additionally, it is important to note that patients often need to further discuss ethical issues that clinicians/researchers may feel have already been resolved. Having a medical ethicist present during these discussions at PPI research meetings may be advantageous.

Furthermore, trust is rarely built in one session, and therefore, it is recommended that multiple meetings, with adequate time between the meetings, be incorporated into the team strategy. The time between meetings allows for all parties to debrief and reflect. In a past evaluation of the PES, it was noted that most researchers simply come to the PES one time even though they are encouraged to return throughout the life of their project. However, the Embryo+™ researchers engaging with the PES more than one time within quick succession showed the PESMs that their time and input were truly valued. Additionally, while building trust is essential for meaningful PPI, [2, 9, 24] examples of how to build trust are not always evident. Here, the PESMs determined that the researchers changing their language and incorporating terminology suggested by the PESMs, using open body language during the meetings, and returning to meet with the PES within a short amount of time specifically led to increased trust between researchers and the PESMs.

Though the current evaluation of this project appears to indicate that the Embryo+™ researchers have been able to meaningfully engage with the PESMs with the assistance of the PES staff, limitations of this evaluation need to be noted. First, the retrospective nature of the discussion groups could potentially lead recall bias within the PES. Additionally, as the PPI evaluation was planned after engagement had occurred, searching for valid and reliable ways to assess the engagement as this stage of the project was difficult as has been previously noted in the literature [25]. While following the GRIPP2 checklist [14] for reporting and the CORE measures [9] for guidance were helpful, a lack of validated measurement tools was a hinderance, hence the reason for usage our proxy loyalty measure [25].

Though not often practiced in basic science research [2], this evaluation shows that PPI can be meaningfully incorporated into these types of scientific studies. Additionally, patients can often identify and discuss social justice and ethical issues in scientific studies, which could be beneficial to basic science researchers who rarely intersect with these topics in their research. Further research evaluating PPI in bench science is needed.

In response to these lessons learned and the limitations of the project, the PES has implemented changes which may also assist others when planning PPI evaluations:

1. The director has increased PPI training and discussion with researchers during their initial meetings to highlight the importance of building trust with the PESMs during meetings. Additionally, if researchers indicate that ethical issues are not of a concern and not needed to be discussed, the Director will probe more deeply to inquire as to why the research teams do not want to engage in ethical discussions. The Director will also clarify that PPI initiatives can provide extensive and valuable feedback on ethical issues in research and may assist in the translation of research into practice.
2. A more rigorous approach to data collection is being implemented for future engagement evaluations.
  - Prior to this project, the PES staff would only evaluate the PES program as a whole and generally not invest resources for the evaluation of individual projects. This was mainly due to the fact that most research teams have not worked the PES throughout the full course of their projects, they engage with the PES at the beginning and not many have returned for follow up meetings.
  - Logic models are being created to help guide the future evaluation of individual projects. This will include follow up with research teams after the meetings as well as a short evaluation survey sent to PESMs after the meetings.
  - Work towards a validation of our Loyalty measure to see if it does show reliably measure meaningful engagement.
3. The PES is continuing to look for partnerships with bench science researchers to help further our understanding of how the PESMs can feel that the engagement they have participated in has been meaningful.

## Conclusions

We described PPI activities in the beginning stages of a research project and evaluated the PESMs’ engagement in a basic science study seeking to improve IVF outcomes. The PES developed the methodology for selecting donors and provided input into potentially upsetting language researchers use that could hinder patients’ acceptance of the research. Future research will explore the impact of PPI throughout the final stages of the Embryo+™ study. This evaluation demonstrates the impact patients can have by providing input into ethically charged basic science research studies.

## Supplementary Information

**Additional file 1.**

**Additional file 2.**

**Additional file 3.**

## Data Availability

The datasets used and/or analyzed during the current study are available from the corresponding author on reasonable request.

## Abbreviations

ART: Assisted reproductive technologies
CMV: Cytomegalovirus
CORE: Critical Outcomes of Research Engagement
IVF: In vitro fertilization
IVF-ET: In vitro fertilization-embryo transfer (IVF-ETs)
PPI: Patient and Public Involvement
PES: Patient Engagement Studio
PESMs: Patient Engagement Studio Members
