# Supplementary material for: Patient engagement in fertility research: bench research, ethics, and social justice
Source: Res Involv Engagem. 2021 May 12;7:29. doi: 10.1186/s40900-021-00278-x (PMC8115861; doi:10.1186/s40900-021-00278-x)
Supplement: Supplementary file 1 — Additional file 1. [file 40900_2021_278_MOESM1_ESM.pdf]

Presentation to the UofSC Patient Engagement Studio Template Guidelines

- This template is to be used as a guide for the **<u>content</u>** of your presentation to the Patient Engagement Studio – this presentation should **NOT** be like other research presentations – remember your audience – these are lay people/patients
- Please feel free to use your own style template – add color, change fonts, add your logos
- The template was created by the patient experts within the Studio to provide you with the best feedback possible.
- Consider the questions on the slides as you are preparing your presentation
- This template is broken into sections based upon the phase of your project, please select the correct section for your presentation.
- If you have further questions please contact the Studio Director, Ann Blair Kennedy, DrPH kenneda5@greenvillemed.sc.edu

UofSC™

Planning the Study/Innovation

Presentation to the University of South Carolina Patient Engagement Studio

[insert project title]

[Date of presentation]

[Project Presenters]

Clarifying Expectations/Purpose

- Why you coming to the Patient Engagement Studio?
- How can we help you?
- What are your expectations?
- What do you need answered/help with?

The Problem [1 or 2 slides]

- What is the problem you are addressing?
- Why are you addressing it?

Approach [2-3 slides maximum]

- How are you planning to address the problem? (Methods of study or plan for program or innovation)

Anticipated impact

- Your “So What” and “Now What”
  - What is the plan to do with the results?

Questions for the PES [*How can we help at this stage of the project?*]

- Items the patient engagement studio can specifically assist with:
  - Planning the study
    - Clarifying your research question
    - Assisting in helping to determine study/innovation participant characteristics
    - Minimizing disruptions to patients
  - Conducting the study
    - Study materials and protocols
      - Incentives
      - Data sharing
    - Patient recruitment
    - Study implementation
    - Data interpretation
    - Patient engagement throughout the life of the study

Project/Innovation Investigators and Leaders

- Please list the names of those involved in the project, their role(s) in the project, their title(s), their institution(s), and their contact information
  - Example: Ann Blair Kennedy, DrPH, Primary Investigator, Clinical Assistant Professor, UofSC SOM Greenville, Kenneda5@greenvillemed.sc.edu

Conducting the Study

Presentation to the University of South Carolina Patient Engagement Studio

[insert project title]

[Date of presentation]

[Project Presenters]

Clarifying Expectations/Purpose

- Why you coming to the Patient Engagement Studio?
- How can we help you?
- What are your expectations?
- What do you need answered/help with?

The Problem [1 or 2 slides]

- What is the problem you are addressing?
- Why are you addressing it?

- If you have presented this project to the studio previously – a brief reminder of the project is all that is needed. A short summary

Approach [1-2 slides maximum]

- How are you currently addressing the problem? (Methods of study or brief description of the program or innovation)

Areas of Concern

- *This slide is only necessary if you are having specific areas of concern with your project as it is being implemented.*
  - Example: Are you having trouble with participant recruitment or attrition from the study?

Findings

- *This slide is used when you are asking the Studio for assistance in data interpretation, further engagement issues, and next steps for the project*
- What did you discover?

Next Steps

- Your “So What” and “Now What”
  - What is the plan to do with the results?

Questions for the PES [*How can we help at this stage of the project?]*

- Items the patient engagement studio can specifically assist with at this stage of your project:
  - Conducting the study
    - Attrition and recruitment issues
    - Study implementation
    - Data interpretation
    - Patient engagement throughout the life of the study
  - Dissemination of Results
    - Identification of patient organizations
    - Planning for dissemination other than academic journals and conferences
    - Suggestions for how to include patients in the dissemination
    - Helping to identify other opportunities for sharing information about the study

Project/Innovation Investigators and Leaders

- Please list the names of those involved in the project, their role(s) in the project, their title(s), their institution(s), and their contact information
  - Example: Ann Blair Kennedy, DrPH, Primary Investigator, Clinical Assistant Professor, UofSC SOM Greenville, Kenneda5@greenvillemed.sc.edu

Dissemination of Results

Presentation to the University of South Carolina Patient Engagement Studio

[insert project title]

[Date of presentation]

[Project Presenters]

Clarifying Expectations/Purpose

- Why you coming to the Patient Engagement Studio?
- How can we help you?
- What are your expectations?
- What do you need answered/help with?

The Problem [1 or 2 slides]

- What is the problem you are addressing?
- Why are you addressing it?

- If you have presented this project to the studio previously – a brief reminder of the project is all that is needed. (A short summary)

Approach [1-2 slides maximum]

- How are you currently addressing the problem? (Methods of study or brief description of the program or innovation)

Approach [1-2 slides maximum]

- How are you currently to addressing the problem? (Methods of study or brief description of the program or innovation)

Findings

- What did you discover?
- If you have previously presented, how have you incorporated feedback from the Studio into your project?

Next Steps

- Your “So What” and “Now What”
  - What is the plan to do with the results?

Questions for the PES [*How can we help at this stage of the project?*]

- Items the patient engagement studio can specifically assist with at this stage of your project:
  - Dissemination of Results
    - Identification of patient organizations
    - Planning for dissemination other than academic journals and conferences
    - Suggestions for how to include patients in the dissemination
    - Helping to identify other opportunities for sharing information about the study
  - Planning the NEXT study
    - Clarifying your research question
    - Assisting in helping to determine study/innovation participant characteristics
    - Minimizing disruptions to patients

Project/Innovation Investigators and Leaders

- Please list the name of those involved in the project, their role in the project, their title, their institution, and their contact information
  - Example: Ann Blair Kennedy, DrPH, Primary Investigator, Clinical Assistant Professor, UofSC SOM Greenville, Kenneda5@greenvillemed.sc.edu

Patient Engagement Studio—Feedback Report

[Title of Presentation]

Investigators
[insert list of investigators with * for those who were present]
----
*Present

[Provide a brief overview/summary of presentation]

The remainder of this report is presented in a manner that corresponds to recommendations for patient engagement made by PCORI. The report includes notes and comments from the studio session as well as additional comments provided by patient experts after the studio meeting.

PLANNING THE STUDY

CLARIFYING THE RESEARCH QUESTION: The patient experts, hereafter referred to as experts, seemed to clearly understand [or not understand depending on reaction] the scope of the project. [Briefly describe the scope of project and/or the research question. Include any pertinent patient feedback on the specific research question]

PARTICIPANT CHARACTERISTICS: [Provide information presented on who will be the study participants and the patient experts suggestions and questions on the topic]

MINIMIZING DISRUPTIONS TO PATIENTS: The following questions/statements were brought up by the experts during the discussion specifically addressing issues that may involve disrupting the lives of patients and/or patient safety. [provide questions/statements/resolution to questions]

CONDUCTING THE STUDY

STUDY MATERIALS AND PROTOCOL: The study staff described the following materials that will be included in the study: [This section should include any research protocols, testing, procedures, informed consent or other documents, etc that study participants will have to interact with as reported by the researchers. Summarize the Patient experts' comments/questions/suggestions on these topics.]

PATIENT RECRUITMENT: [Describe how participants will be recruited into the study and the Patient Experts comments/questions/suggestions on these topic.]

STUDY IMPLEMENTATION: [How are the research staff going to monitor study implementation and keep study participants in the study (reduce attrition and/or increase adherence to study protocol). Not all presentations will discuss this clearly. If it is not discussed, simply put something like – the monitoring of the implementation was not discussed.]

DATA COLLECTION AND ANALYSIS: [Provide description on how researchers plan to collect and analyze the data including the patient experts feedback – this could include review of a survey and or interview questions, processes for data collection, and/or interpretation of analyzed data. In some presentations, the patient experts will be asked to provide interpretation of data and this section may be the largest in the report – see the Petrizzo report as an example]

ENGAGMENT: [Describe how the researchers planning on keeping patient experts engaged throughout the life of the project. If it is not discussed can put something like - Additional patient engagement efforts during the study were not discussed but it is suggested that the researchers return to the Patient Engagement Studio to discuss XYZ.]

DISSEMINATION OF RESULTS

[We haven't had many discuss dissemination of results yet and this is what we have written for those who have not discussed dissemination. Plans for dissemination (PCORI content areas below) were not discussed in this review. The researchers, if interested, are welcome to return to discuss these areas once the study results are known. Local dissemination could be easily employed. A few standard suggestions by the Studio Director are listed below as we like to encourage investigators to disseminate successful results broadly and to patients when possible.]

IDENTIFICATION OF PATIENT ORGANIZATIONS FOR DISSEMINATION. [If this was not discussed – provide a list of some groups to send the info to]

PLAN FOR DISSEMINATION. [If there is no discussion on this point – the following can be added - Other than .disseminating though the general academic process of publication in a journal and or presentations at a conference; we recommend that other types of dissemination be considered that may get results into the hands of patients more quickly particularly patients within the Prisma Health system. Develop a plan beyond simple publication in scientific journals and conferences, ask patients for questions.]

PARTICIPATION IN DISSEMINATION. [If there is no discussion on this point – the following can be added -Include patients when disseminating results, let them tell about their experiences, perhaps include them in reaching other patients via an additional "testimonial" video.]

IDENTIFYING OPPORTUNITIES FOR SHARING INFORMATION ABOUT THE STUDY. [If there is no discussion on this point – the following can be added Link with the other studies who utilized the PES to find common ground for sharing.]

Summary report prepared by [names]
[Date finalized]
