# Supplementary material for: Patient engagement in fertility research: bench research, ethics, and social justice
Source: Res Involv Engagem. 2021 May 12;7:29. doi: 10.1186/s40900-021-00278-x (PMC8115861; doi:10.1186/s40900-021-00278-x)
Supplement: Supplementary file 2 — Additional file 2. [file 40900_2021_278_MOESM2_ESM.pdf]

Appendix 1. Survey sent to PES members who participated in the Embryo+ Project

Patient Engagement with Embryo+

Start of Block: Default Question Block

We are gathering just a bit of information from you to add to our Embryo+ patient engagement evaluation project.

This survey should take approximately 5-10 minutes to complete. Please answer each of the following questions to the best of your ability. If you are not sure of an answer to a question, please provide your best estimate. Your responses will be kept confidential.

Are you willing to participate?

- ○ Yes (1)
- ○ No (2)

Skip To: End of Survey If Are you willing to participate? = No

Did you attend at least one of the two Embryo+ presentations to the PES?

- ○ Yes (1)
- ○ No (2)

Page Break

End of Block: Default Question Block

Start of Block: Block 1

We are interested in gathering just a little more information about your participation in the Embryo+ project with the Patient Engagement Studio.

This survey has some questions ask you to rate your level of agreement in response to a statement and others ask for you to provide your comments and feedback. You are encouraged to share your experiences and any information you feel is relevant and will help us improve our engagement processes.

We appreciate your feedback. It is critical to helping us improve our engagement processes. Please be aware that all of the information you provide will be confidential.

Thank you for your participation!

We will start with some basic demographics

What is your gender?

- Man (1)
- Woman (2)
- Nonbinary (3)
- Transgender man (4)
- Transgender woman (5)
- Gender queer (6)
- Bigender (7)
- Two-spirit person (8)
- Another, not listed (9)
- Choose not to answer (10)

Display This Question:

If What is your gender? = Another, not listed

You answered "Another, not listed" with regards to your gender, please describe

____

What is your age (in years)?

____

What is your race?
Please check all that apply

- ☐ Native American or Alaskan Native or Indigenous person (1)
- ☐ Asian (2)
- ☐ Black or African American (3)
- ☐ Native Hawaiian or Pacific Islander (4)
- ☐ White (5)
- ☐ Another, not listed (6)
- ☐ Choose not to answer (7)

Display This Question:

If What is your race? Please check all that apply = Another, not listed

You answered "Another, not listed" with regards to your race, please describe

____

What is your ethnicity?

- ○ Hispanic or latino/a/x (1)
- ○ Non-hispanic or latino/a/x (2)

What is your religious identity?

- ◯ Agnostic (1)
- ◯ Atheist (2)
- ◯ Baha'i (3)
- ◯ Buddhist (4)
- ◯ Catholic (5)
- ◯ Hindu (6)
- ◯ Jewish (7)
- ◯ Mormon (8)
- ◯ Muslim (9)
- ◯ Protestant (10)
- ◯ Sikhism (11)
- ◯ Another, not listed (12)
- ◯ Choose not to answer (13)

Display This Question:

If What is your religious identity? = Another, not listed

You answered "Another, not listed" with regards to your religious identity, please describe

____

What is your highest level of education?

- ○ Less than high school (1)
- ○ High school graduate (2)
- ○ Vocational/technical school (3)
- ○ Some college (4)
- ○ Associates degree (5)
- ○ Bachelors degree (6)
- ○ Some graduate school (7)
- ○ Masters degree (8)
- ○ Doctorate/Professional degree (9)

Page Break

Which of the following PES role(s) did you fill when attending the Embryo + meetings? (Check all that apply)

- ▢ Patient Expert (1)
- ▢ Clinician (2)
- ▢ Community member (3)
- ▢ Community partner (4)
- ▢ Staff member (5)
- ▢ Researcher (6)
- ▢ Another, not listed (7)

Display This Question:

If Which of the following PES role(s) did you fill when attending the Embryo + meetings? (Check all... = Another, not listed

You answered "Another, not listed" with regards to your role(s) when attending the Embryo+ meetings, please describe

____

Which of the Embryo+ meetings did you attend?
Check all that apply

- ☐ Embryo+ meeting 1 (project introduction) (1)
- ☐ Embryo+ meeting 2 (selecting donors) (2)
- ☐ Discussion about meeting 1 (med student led meeting) (3)
- ☐ Discussion about meeting 2 (med student led meeting) (4)

Page Break

These last few questions about concerning your perceptions of your engagement with the Embryo+ project

Q19 Do what level do you agree or disagree with the following statement.

I am satisfied with my participation with the Embryo+ project

- Strongly disagree (1)
- Disagree (2)
- Somewhat disagree (3)
- Neither agree nor disagree (4)
- Somewhat agree (5)
- Agree (6)
- Strongly agree (7)

Page Break

Do what level do you agree or disagree with the following statement.

Engagement with the Embryo+ project is a good use of my time.

- Strongly disagree (1)
- Disagree (2)
- Somewhat disagree (3)
- Neither agree nor disagree (4)
- Somewhat agree (5)
- Agree (6)
- Strongly agree (7)

Page Break

How likely are you to continue working with the Embryo+ project if they return to the PES?

- ○ Extremely unlikely (1)
- ○ Moderately unlikely (2)
- ○ Slightly unlikely (3)
- ○ Neither likely nor unlikely (4)
- ○ Slightly likely (5)
- ○ Moderately likely (6)
- ○ Extremely likely (7)

Page Break

How likely are you to recommend that others join the Embryo+ project as a patient expert?

- ○ Extremely unlikely (1)
- ○ Moderately unlikely (2)
- ○ Slightly unlikely (3)
- ○ Neither likely nor unlikely (4)
- ○ Slightly likely (5)
- ○ Moderately likely (6)
- ○ Extremely likely (7)

Page Break

How likely are you to recommend that others join the Embryo+ project as a study participant?

- ○ Extremely unlikely (1)
- ○ Moderately unlikely (2)
- ○ Slightly unlikely (3)
- ○ Neither likely nor unlikely (4)
- ○ Slightly likely (5)
- ○ Moderately likely (6)
- ○ Extremely likely (7)

Page Break

How valuable do you find your participation with the Embryo+ project?

- ◯ No value (1)
- ◯ Very low value (2)
- ◯ Low value (3)
- ◯ Fair amount of value (4)
- ◯ Good value (5)
- ◯ Very good value (6)
- ◯ Excellent value (7)

What else would you like us to know about your experience with the Embryo+ project?

____

____

____

____

____

End of Block: Block 1
