# Supplementary material for: Patient engagement in fertility research: bench research, ethics, and social justice
Source: Res Involv Engagem. 2021 May 12;7:29. doi: 10.1186/s40900-021-00278-x (PMC8115861; doi:10.1186/s40900-021-00278-x)
Supplement: Supplementary file 3 — Additional file 3. [file 40900_2021_278_MOESM3_ESM.docx]

Appendix C: Discussion Group Questions Guide

| Questions | GRIPP2 Topic (If Applicable) |
| --- | --- |
| 1. What do you remember about the meetings? | 4a |
| 2. What was your opinion of the researchers? Were they receptive of your role? Do you feel like they listened to you? What did they do that made you feel like your input was valuable? | 8g |
| 3. Do you believe it beneficial to attend both meetings? If you missed the first meeting, did you feel adequately prepared for the second meeting? | 4c |
| 4. What does PPI mean to you? What do you see as the role of PPI? How do you feel like you contributed to this project? How would you sum up the role of PES with this project? | 2a |
| 5. If you attended both meetings, were there any change in what you thought from one meeting to the next? Any confusion or concerns that were cleared up? |  |
| 6. In your own words, what was the goal of Embryo+™ coming to the PES? | 3 |
| 7. How did you see your role in determining the pricing structure? Were you concerned you would price people out of Embryos? Are you satisfied with the sliding scale as discussed? | 6 |
| 8. Was there anything that happened during the meeting that you felt helped or hindered the process? | 7d |
| 9. Was it easy to separate the factors you were looking for in a donor from their looks? |  |
| 10. Was there any concern that the IVF clinic being used for the research is not local? |  |
| 11. It seemed like there was a change in concern about ethics between the two meetings. What was that change? What changed your opinion? |  |
| 12. Do you think any of your concerns, especially regarding siblings of IVF children, are based on where you grew up? Where are you originally from, or how long have you been in the local area? |  |
| 13. Would anyone be willing to have individual conversations with us about their experiences? |  |
